# The role of mitochondria in the development and progression of lung cancer

**DOI:** 10.5936/csbj.201303019

**Published:** 2013-12-07

**Authors:** Emily R Roberts, Kelly Jean Thomas

**Affiliations:** a Colorado Mesa University, Biological Sciences Department, 1100 North Ave, Grand Junction, CO 81501, USA

**Keywords:** apoptosis, non-small cell lung cancer, mitochondrial dynamics, mitochondrial dysfunction, tumorigenesis

## Abstract

The influence of mitochondria in human health and disease is a rapidly expanding topic in the scientific literature due to their integral roles in cellular death and survival. Mitochondrial biology and alterations in function were first linked to cancer in the 1920s with the discovery of the Warburg effect. The utilization of aerobic glycolysis in ATP synthesis was the first of many observations of metabolic reprogramming in cancer.

Mitochondrial dysfunction in cancer has expanded to include defects in mitochondrial genomics and biogenesis, apoptotic signaling and mitochondrial dynamics. This review will focus on the role of mitochondria and their influence on cancer initiation, progression and treatment in the lung.

## Introduction

Mitochondria are subcellular, membrane-enclosed, organelles that are essential for cell survival in eukaryotes. These dynamic organelles are fundamental for energy production, providing substrates for intracellular metabolic pathways. Mitochondria also influence cellular signaling and survival pathways, including apoptosis [1]. Mitochondrial dysfunction has been implicated in a plethora of human diseases, most notably in cancer and aging.

Lung cancer is the most common type of cancer diagnosed in the world [2] and is the number one cancer killer in the United States [3]. It accounts for approximately 14% of all cancer diagnoses and 28% of cancer-attributed deaths in Americans [3]. Survival rates among patients with lung cancer are much lower than survival rates for patients with other common cancers, such as breast, colon or prostate cancer. The five-year survival rate for early stage lung cancer is 52.6% and for metastatic lung cancer is only 3.5%; approximately half of all patients die within a year of their initial diagnosis [4]. Screening options for lung cancer are very limited - only 15% of cases are diagnosed at an early stage [4]. Additionally, treatment is often ineffective, as evidenced by low survival rates, and costs an estimated 10.3 billion dollars in the United States annually [5]. This review will highlight the roles of mitochondrial-mediated apoptosis in lung cancer and summarize the potential impact of mitochondrial dysfunction on tumorigenesis, including the contribution of mitochondrial dynamics to lung cancer progression. Currently, the mitochondria are viable targets for both diagnostic screening and therapeutics and further understanding of their contribution to cancer initiation, progression and treatment is essential.

## Mitochondrial dysfunction in lung cancer: Altered bioenergetics and genomic instability

Mitochondria possess their own double-stranded circular genome, encoding 13 genes whose protein products are subunits of the respiratory chain or the oxidative phosphorylation system (OXPHOS). Mitochondrial DNA (mtDNA) is present in high copy numbers (10^3^-10^4^ copies per cell) in virtually all cells. Electron transport and ATP synthesis by oxidative phosphorylation act continuously within a mitochondrion. The electron transport chain (ETC), organized into five separate enzymes (Complexes I-V), resides in the inner mitochondrial membrane and is the common pathway by which electrons, derived from energy rich molecules, flow to oxygen. Complexes I through IV each contain part of the electron transport chain, whereas Complex V catalyzes the synthesis of ATP. The final acceptor of the electrons is oxygen, which is reduced to water by the addition of four electrons.

During mitochondrial respiration (most notably by Complexes I and III), toxic superoxide anion radicals are deleteriously produced when electrons are captured by oxygen. These superoxide radicals (O_2-•_) are converted to hydrogen peroxide (H_2_O_2_) by the mitochondrial enzymes manganese superoxide dismutase, SOD2 (matrix localization) and cooper/zinc superoxide dismutase, SOD1 (intermembrane space localization). Within the mitochondria hydrogen peroxide is broken down to water by the action of glutathione peroxidases (GPX) or peroxiredoxins. In addition to the antioxidant enzymes mentioned above, cells have nonenzymatic scavengers (glutathione, vitamins E and C, ubiquinone) to protect against reactive oxygen species (ROS). Complexes I, II and III subunits are all capable of generating ROS during OXPHOS and oxidative stress is the result of an imbalance between ROS production and antioxidant action. During conditions of high radical production or low antioxidation, these ROS can affect cell integrity, which can damage macromolecules such as lipids, proteins and DNA. The Complex I subunit has a special significance in tumor progression and metastasis. If Complex I is mutated, it will not interact with Complex III in supercomplexes, which results in the incorrect number of electrons being transferred and, eventually, ROS overproduction cannot be detected by these cells. This results in an overproduction of ROS, increased energy loss, and oxidative stress [6]. In lung cancer, Complex I frequently appears to be altered, especially in patients that have never smoked. Forced over expression of mutated Complex I in mitochondria increased invasion, superoxide particles, and proliferation *in vitro* [7].

Mitochondrial dysfunction, including metabolic alterations, has been observed in cancer cells. For instance, many tumor cells exhibit excessive glucose conversion to lactic acid in the presence of oxygen (aerobic glycolysis) as well as concomitantly suppressed mitochondrial respiration, a phenomenon known as the Warburg effect [8]. The Warburg effect in cancer cells demonstrates that mitochondria are dysfunctional by utilizing glycolysis to generate ATP. Despite oxygen being present, tumor cells prefer to generate ATP rapidly via glycolysis, which produces 16-fold less ATP than OXPHOS, but facilitates the increased rates of cell growth and proliferation [9]. When the balance between glycolysis and oxidative phosphorylation is disrupted, mitochondrial metabolites accumulate in the cytoplasm and influence metabolism. A side effect of such an imbalance is hypoxia or low oxygen levels. Unbridled cell proliferation within a tumor inevitably outgrows its blood supply, which lowers oxygen availability and cells enter a hypoxic state. These hypoxic conditions are thought to drive cancer progression by promoting genomic instability [10]. In the A549 lung cancer cell line, mitochondria are enlarged when in a hypoxic state; enlarged mitochondria appear to be resistant to apoptosis and thus allow cancer cells to continue proliferating [11]. Hypoxic conditions stabilize the hypoxia-inducible transcription factor (HIF-1α), which facilitates cells adapting to stressful environments by transcribing and synthesizing ∼70 hypoxia-related factors [12]. HIF-1α and other factors such as mutations in oncogenes, tumor suppressors and/or signaling kinases help to regulate glycolytic conversion and compensate for low-ATP yields in cancer [13–16]. When cells are stressed for energy sources, AMPK (5’ adenosine monophosphate-activated protein kinase) is activated due to an increase in the AMP/ATP ratio. This inhibits cell proliferation and the cell begins making energy via oxidative metabolic pathways [17–19]. In order to grow, tumor cells suppress AMPK signaling by altering signaling pathways and oncogenic mutations [19]. AMPK suppression allows tumor cells to grow under abnormally low nutrient conditions and cells proliferate [19], bypassing typical growth checkpoints via oncogene expression and loss of tumor suppressing genes (such as *p53)*. The loss of AMPK signaling and the cascade of effects thereafter contribute to tumor growth and likely to energy production using glycolysis [20]. For example, the oncogene, *c-myc*, is overexpressed in 20% of cancers and contributes to tumor cell proliferation [21], enhances glycolysis and its production of precursors [12, 22] and coordinates the regulation of metabolic networks to allow rapid entry into the cell cycle [23]. Additionally, the tumor suppressor *p53*, which is mutated or deleted in 50% of solid tumors [24], no longer induces cell-cycle arrest, apoptosis, DNA repair or senescence in response to cellular stress. Lack of p53 function has been found to be advantageous for tumor cells by downregulating hypoxia-induced apoptosis [24–27] and interfering with ETC assembly and promoting a shift in metabolism to a more glycolytic state [28]. As such, killing lung cancer cells using a glycolytic inhibitor is more efficient in cells without functional p53 [29]. The loss of tumor suppressors (p53) or the activation of oncoproteins effect the onocogenic signaling pathways, thus impacting cancer cell growth and survival [30]. Additional changes in metabolism are influenced by activation of Akt (acute transforming retrovirus thymoma protein kinase), which is often observed in cancer cells [12]; in fact, many studies point to the contribution of deregulated Akt in the development or progression of lung cancer [31]. For example, increased glycolytic capacity caused by complex transcriptional changes, such as inhibition of the forkhead box subfamily O (FOXO) transcription factors, is associated with changes in Akt1 signaling and cellular transformation processes [30]. However, current debates in the field address whether changes in metabolism are a cause or consequence of neoplastic transformation [12].

Mitochondria are continuously exposed to mutagenic oxygen radicals generated by OXPHOS [32]; consequently, oxidative stress can alter the structure of the respiratory chain and cause proton leakage, mitochondrial uncoupling and mitochondrial DNA damage [33]. As such, mitochondrial DNA (mtDNA) acquire 10-fold more mutations than nuclear genomic DNA primarily because the mitochondrial genome lacks protective histones, introns and efficient DNA repair systems. Additionally, antioxidant defense mechanisms that protect the cell from damaging reactive oxygen species (ROS) are deficient in tumor cells [34, 35]. Persistent oxidative stress in cells promotes DNA damage and subsequent cancer growth and metastasis. As such, mutations in mtDNA have been reported in human cancers [36–41]. Alterations of the non-coding displacement (D) loop of mtDNA are present in many cancers [42]. Fliss et al analyzed mutations in mtDNA in bladder, head and neck, and lung cancer and found mutations in 50% of cancers of which 67% were in the mtDNA D-loop region, suggesting a veritable genomic instability hot-spot [38]. Significantly elevated mutation rates in the mtDNA D-loop region were observed in exhaled breath condensate of patients with lung cancer when compared to non-diseased controls; it has been proposed that mtDNA mutations may be a marker of carcinogenesis of the lung [43].

In addition to mtDNA mutation, mtDNA content is positively associated with the risk of lung cancer [44, 45]. It has been suggested that mtDNA content is thought to increase during tumorigenesis to compensate for mitochondrial dysfunction or damage [45]. However, it is unclear whether these alterations contribute to tumorigenesis or are a secondary consequence of the carcinogenic process. To better address this issue, studies utilizing techniques in next-generation sequencing for the rapid-high-throughput detection of mtDNA mutations or mtDNA content have allowed for examination of various tissues and body fluids in cancer patients to help address the timing of mtDNA alterations in the course of disease development [46]. As, such mitochondrial mutations and content are being explored as molecular markers of cancer detection and progression.

Mitochondrial DNA is also susceptible to damage by environmental carcinogens, such as smoking [46]. Inequality between the frequency and nature of mtDNA mutations between smoker- and non-smoker lung cancer patients has recently been revealed to expand upon the impact of smoking on lung tumorigenesis. With no associations observed between mtDNA mutations with neither age nor gender, the frequency of mtDNA mutation was significantly higher in non-smokers when compared to smokers [7]. This smoking-independent pathway for acquiring mtDNA mutation gives insight into understanding the mitochondrial genetic alterations of these more common new lung cancer cases being seen in clinical settings. However, the majority of the coding mtDNA mutations identified targeted complex I of the respiratory chain and ectopic expression of these mutations increased cellular proliferation, invasion and ROS production in lung cancer cells *in vitro* [7]. To support this observation, Complex I (NADH dehydrogenase) activity has been shown to be decreased in cells with high metastatic potential [47].

Furthermore, complex I deficiencies alter mitochondrial morphological phenotypes [48], which are indicative of mitochondrial health (as will be discussed later in the review), and influence the initiation of apoptosis - another key function of mitochondria, which is to control programmed cell death. Additionally, mitochondrial permeability transition (MPT) is the sudden permeabilization of the inner mitochondrial membrane in response to stimuli such as increased ROS production or hypoxia that can lead to mitochondrial swelling and apoptotic cell death. The mitochondrial permeability transition pore complex (PTPC) is a multimeric protein complex that spans both mitochondrial membranes and contains the following proteins: voltage-dependent anion channel (VDAC), adenine nucleotide translocase (ANT), cyclophilin D (CYPD), and peripheral benzodiazepine receptor (PBR) [49]. Alterations of the PTPC and its influence on apoptotic circumvention in lung cancer will be discussed in the next section.

**Figure 1:**
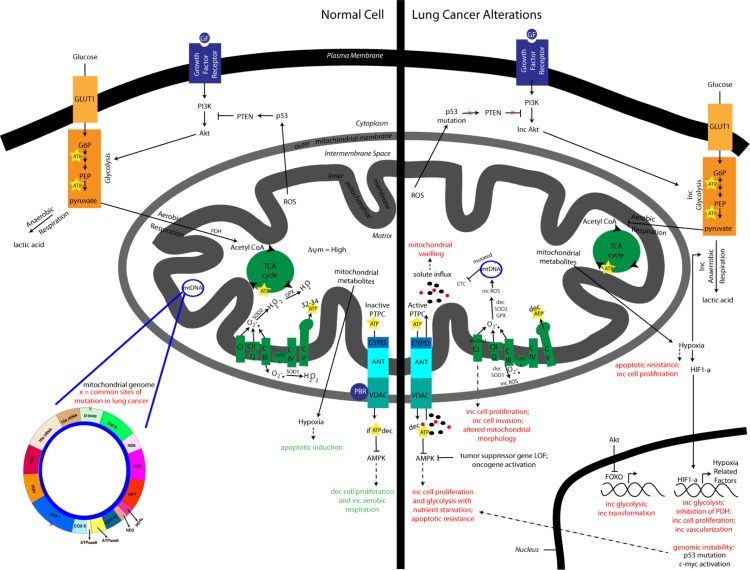
**Altered bioenergetics and genomic instability in lung cancer.** This comparison shows lung cancer alterations in bioenergetic signaling pathways, including a shift towards the reduced production of ATP using glycolysis instead of oxidative phosphorylation and the consequential impact on cancer initiation and progression. Additionally, mitochondrial dysfunction that occurs as a by-product of altered bioenergetics is displayed showing how genomic instability promotes tumorigenesis.

Figure 1 illustrates the bioenergetic alterations and genomic instability that occurs in lung cancer as a consequence of mitochondrial dysfunction.

## Mitochondrial-mediated apoptosis and lung oncogenesis

Apoptosis is an evolutionarily conserved multistage process that maintains homeostasis in adult tissues [50]. Cancer cells have a variety of ways of preventing apoptosis, such as the mutation of apoptotic-promoting proteins, anti-apoptotic proteins, and mitochondrial dysfunction [6]. Mitochondrial DNA mutations also appear to allow cancer cells to evade apoptosis [51]. A classic cancer hallmark proposed by Hanahan and Weinberg is apoptotic resistance. It is important to understand the molecular events that contribute to drug-induced apoptosis and how tumors evade apoptotic cell death as many cancer treatments attempt to induce apoptosis in cancer cells but activation of the apoptotic machinery is not always effective.

Defects in apoptosis are implicated in both tumorigenesis and drug resistance, and these defects are one cause of chemotherapy failures. The type of cancer determines its sensitivity to apoptosis. For example in lung cancer, when compared to non-small cell lung carcinoma (NSCLC), small cell lung carcinoma (SCLC) lines were more prone to spontaneous apoptosis [52]. Consequently, the absence of spontaneous apoptosis and ineffective treatment-induced apoptosis in non-small cell lung carcinoma suggests that deficiencies in the apoptotic process may be responsible for their resistance to anti-cancer therapy.

Similar to other types of cancer, gene mutations (ie., *p53, c-myc, Akt*) and altered expression of apoptosis regulators are also detected in lung cancer. It has been suggested that different sensitivity to therapeutics that induce apoptosis may be related to the expression of apoptosis regulators in lung cancer [53]. Caspases, a family of cysteine proteases, are the central regulators of apoptosis. They mediate both forms of apoptosis--the intrinsic (which is activated by stress and controlled by the mitochondria) and extrinsic (which is activated by the death receptor pathway) apoptotic signaling cascades. Inactive pro-caspases are activated by cleavage cascades that signal to trigger downstream effectors, which cleave cellular substrates to induce apoptosis [54]. For example, studies show that small cell lung carcinoma cells, which are more prone to spontaneous apoptosis, are deficient in mRNA expression of extrinsic pro-caspases-1, -4, -8, and -10 [52] but no differences were observed between NSCLC and SCLC after examining the expression of intrinsic pro-caspases-2, -3, -6, -7, -9 [55]. These data suggest that regulation or execution of caspases may contribute to apoptosis propensity.

Inhibitors of apoptosis proteins (IAPs) prohibit cell death by binding and inactivating executioner caspases. IAPs work downstream of both mitochondria-mediated and death receptor-mediated apoptotic pathways. A study examining a panel of NCI human lung tumor cell lines detected overexpression of XIAP and CIAP1 at the mRNA and protein level [56]. Additionally, the IAP-family protein Survivin, was highly overexpressed in lung tumor cell lines [57] and lung tumors [58]. Survivin has been shown to inhibit not only apoptosis induced by death receptor activation via FADD (Fas-associated protein with death domain) but this IAP can inhibit programmed cell death due to Bax overexpression, which engages the apoptotic pathway at the level of mitochondria by inducing cytochrome c release [59]. It has been suggested that higher levels of Survivin protein are associated with more aggressive disease [60]. Furthermore, Survivin antisense molecules have been utilized clinically to sensitize lung cancer cells to chemotherapy [61].

The Bcl-2 family of proteins, which regulates apoptosis at the mitochondrial level [62], has a cell death dichotomy - Bcl-2, Bcl-xL, Bcl-w and Mcl-1 family members inhibit apoptosis whereas Bax, Bak and Bok activate apoptosis. In multiple cancers, including certain lung cancers, anti-apoptotic proteins were amplified, suggesting that malignant cells could evade apoptosis via Bcl protein overactivation, signaling the cell to avoid cell death [63]. Cytochrome c release and apoptosis is initiated when BH3-only domain proteins, Puma and Bim, bind and inhibit pro-survival Bcl-2 family proteins. This protein inhibition allows pro-apoptotic Bax and Bak to permeabilize the outer mitochondrial membrane (MOMP) inducing apoptotic events through oligomerization [64]. Resistance to apoptosis has been observed in cells lacking Bim, Puma, and Bid, indicating that these proteins are needed to activate Bax and Bak to signal apoptosis [65]. In non-small cell lung cancer, anti-apoptotic Bcl-2 protein expression was higher in squamous cell carcinomas when compared to adenocarcinomas [66]. Furthermore, Bcl-2 protein expression correlates with nodal status in NSCLC and Bcl-2 immunostaining is considered a marker of loco-regional invasivity [67]. It has been shown that Bcl-2 protein expression is higher in SCLC than NSCLC [66]; however, another study demonstrated the development of SCLC was independent of Bcl-2 expression [68]. Bcl-2 family members control PTPC function [49]. Bcl-2 acts to keep the PTPC inactive through its interaction with VDAC. When PTPC is activated, Bax associates with VDAC and allows matrix solute influx, including calcium and other ions, which promote mitochondrial permeability transition through depolarization of the mitochondrial membrane. This leads to MOMP and the subsequent release of intermembrane space proteins, such as cytochrome c.

Paradoxically in NSCLC, using immunohistochemical analysis to evaluate Bax expression, there is frequent high expression of pro-apoptotic Bax during neoplastic proliferation. However, no correlation was identified between Bax expression and clinicopathologic parameters (ie., tumor grade, histological type) [69]. Despite this, in lung and other tumor types, there is a trend such that anti-apoptotic proteins are active in early tumor formation while pro-apoptotic proteins are inhibited [70].

Following cytochrome c release, the next step in the intrinsic pathway of apoptosis is apoptosome formation. The apoptosome is a large ternary protein structure that upon formation activates pro-caspase-9, which subsequently activates other caspases triggering apoptosis [71]. Alterations in the expression of proteins that regulate apoptosome formation have also been proposed to play a role in carcinogenesis, including overexpression of HSPs (heat shock proteins). HSPs are a family of conserved proteins that protect the cell and are induced in response to a variety of cellular stressors. Defects in apoptosome formation have been found in lung cancer, including the overexpression of HSPs [72], which has been found to be correlated with poorer clinical outcomes and decreased responsiveness to chemotherapy in other cancer types [73].

The tumor suppressor gene *p53* plays a critical role in not just in cell cycle checkpoints, DNA repair and recombination [74] but also apoptosis [75]. Expression of p53 induces expression of death effector and mitochondrial apoptotic pathways. Defects in the p53 pathway are common in lung cancer. Approximately 50% of all lung cancers exhibit dysfunctional p53 protein with *p53* mutations detected in 50% of NSCLC [76] and 90% of SCLC [77]. In many human cancers, *p53* is present as an inactive gene. This inactive form of *p53* appears to allow tumor cells to avoid cell senescence and continue proliferating [78]. However, no relationship has been identified between the mutational status of *p53* and the susceptibility of either SCLC or NSCLC to undergo apoptosis [55]. Additionally, p53 up-regulated modulator of apoptosis (PUMA) plays a major role in p53 dependent apoptosis [79] and in the absence of PUMA, cells are resistant to apoptosis [80] and carcinogenesis is promoted [81]. In human NSCLC cell lines that harbor wild-type *p53*, it has been shown that a small molecule that disrupts p53 binding, which activates p53 and promotes apoptosis via the induction of downstream target pro-apoptotic BH3-only genes, Noxa and PUMA [82].

Currently, cancer therapeutics target various aspects of the apoptotic pathway. Anti-apoptotic modulating therapy is a promising strategy for cancer drug discovery and many groups are investigating the effects of Bcl-2, Bcl-xL or XIAP inhibition using antisense oligonucleotides and small mimetic molecules. For instance, *in vitro* studies are utilizing a BH3 domain small molecule mimetic that functions as a Bcl-2 inhibitor, which binds with high affinity to anti-apoptotic proteins Bcl-2 and Bcl-xL leaving Bax or Bak oligomers free to permeabilize the outer mitochondrial membrane. This process releases cytochrome c from the mitochondria, activates caspase-3 and promotes cell death [83]. A Bcl-2 mimetic, ABT-737, potentiates anticancer treatments in small cell lung cancer xenograft models [84, 85]. Previous studies have suggested that multiple points in the apoptotic pathway may need to be targeted to effectively induce programmed cell death, such as the IAP Survivin [58], Bcl-2 related protein Mcl-1 (myeloid cell leukemia sequence 1) inhibition or Bak induction [86]. In SCLC and some pulmonary carcinoid tumors, a combination of chemotherapy agents, specifically those that down regulate Mcl-1, and Navitoclax, a drug that inhibits anti-apoptotic Bcl proteins, has had some success decreasing tumor size; patients who received the combination treatment for longer than one year displayed a 22% - 35% decrease in tumor size [87]. Both SCLC and pulmonary carcinoid tumors are characterized by having high expression of Bcl-2 expression and the chemotherapy resistance commonly seen in these types of cancer suggests dependence on anti-apoptotic proteins [87]. Another therapy utilized an EGFR (epidermal growth factor receptor) antagonist, which prevented promotion of Akt survival signaling [88] and inhibited cancer cell proliferation. Conversely, pro-apoptotic modulating anti-cancer drugs are also being studied. Direct activation of executioner caspases like caspase-3 [89] or stimulation of cell death receptors involving the extrinsic apoptotic pathway has induced apoptosis and limited cell growth in lung cancer cell lines [90]. Lastly, gene replacement therapy to restore mutated cancer suppressor gene function is another promising strategy for cancer treatment. Restoration of p53 or inactivation of Akt by *PTEN (phosphatase and tensin homolog)* gene delivery inhibited lung tumorigenesis [91] and suppression of Akt by programmed cell death 4 (PDCD4) induced apoptosis in a mouse model of lung cancer [92].

**Figure 2:**
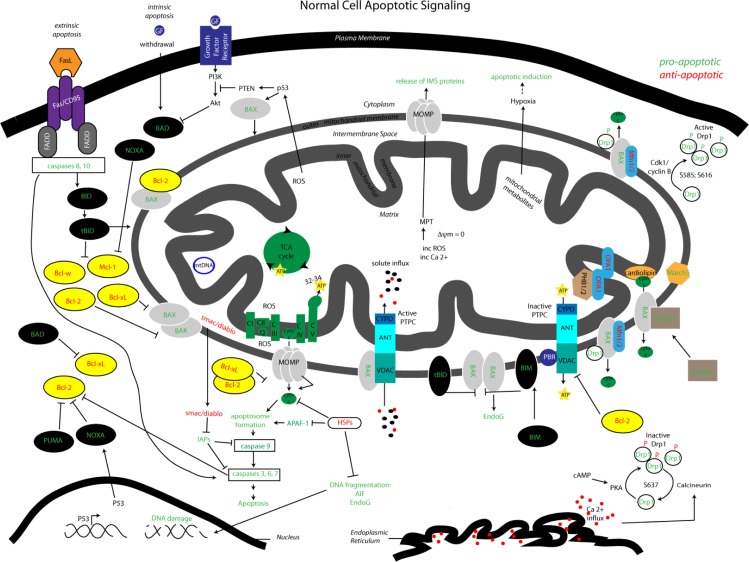
**Mitochondrial pathways: interactions between apoptotic signaling and mitochondrial dynamics.** Visualized is the complex array of proteins and signaling molecules that are involved in normal apoptotic signaling, both extrinsic and intrinsic, and the interaction of mitochondrial dynamics players that modify mitochondrial morphology and consequentially impact apoptotic regulation. Green colored text indicates pro-apoptotic signaling and red colored text indicates anti-apoptotic signaling.

Figure 2 illustrates the intrinsic and extrinsic apoptotic pathways that are involved implicated in lung cancer progression. Impairments in apoptosis play a central role in cancer progression and as such, scientists are exploring the components that engage the cell death machinery to circumvent oncogenesis. An increasing number of studies are investigating the process of mitochondrial dynamics and surveying their role in apoptosis and metabolism.

## Mitochondrial dynamics and tumorigenesis of the lung

The process of mitochondrial dynamics, the continuous cycle of fission and fusion, maintains mitochondrial morphology and function [93]. Solid evidence has shown that mitochondrial dynamics manipulates mitochondrial function including cell death and metabolism [94]. Figure 2 illustrates the complex interaction of mitochondrial dynamics proteins in apoptotic signaling and control of mitochondrial morphology. Defects in mitochondrial dynamics have been recently brought to the forefront of neurodegenerative therapeutic modeling, for diseases such as Parkinson and Alzheimer disease [95]. The number of studies investigating the role of mitochondrial function in cancer is on the rise, but understanding the recent contributions made in the field of mitochondrial dynamics may impact our comprehension of tumorigenesis and cancer progression. If we manipulate mitochondrial dynamics to sensitize tumor cells to apoptosis, then site-directed therapeutics could potentially be applied in cancer.

The mitochondrion plays a complex role during apoptosis. The mitochondrial stage of apoptosis control is upstream of caspase activation and is mediated by the Bcl-2 family of proteins. Upon the initiation of apoptosis, BH3-only proteins are induced and bind anti-apoptotic Bcl-2 proteins, which allow pro-apoptotic Bax and Bak to permeabilize the outer mitochondrial membrane releasing cytochrome c and activating the caspase signaling cascade [96]. Upstream of caspase activation and prior to or during cytochrome c release, a mitochondrion undergoes a division whereby the mitochondrial network segregates into smaller units—this process is called mitochondrial fission [97]. Mitochondria are dynamic organelles that constantly divide and fuse to form a mobile, interconnected network of mitochondrial units. The opposing process of fission is the joining of two mitochondria, mitochondrial fusion, which promotes elongated and reticular mitochondrial phenotypes [98]. Mitochondrial dynamics is the balance between mitochondrial fission and fusion events that regulate the shape, structure and function of the mitochondrion.

Mitochondrial fragmentation or fission is associated with apoptosis; however, excessive mitochondrial fission can occur in the absence of programmed cell death [96, 99]. For instance, reversible uncoupling of the inner mitochondrial membrane by depolarizing agents fragments the mitochondria during treatment. With drug removal the mitochondria repolarize and fuse together to form a healthy network [100]. Excessive mitochondrial fission is essential for intrinsic apoptosis—it is necessary for cytochrome c release and downstream caspase activation [101, 102]. Additionally, mitochondrial fission proteins Drp1 (Dynamin-related protein 1; a large GTPase), Fis1 (an assembly protein for Drp1 mitochondrial recruitment in yeast), MARCH5 (a mitochondrial E3 ubiquitin ligase) and EndoB1 (Endophilin B1; BAR domain-containing protein) have been associated with apoptosis [103–105].

During apoptotic initiation, Drp1 is recruited from the cytoplasm to the outer mitochondrial membrane [101, 106–108], where it colocalizes with pro-apoptotic Bax and mitochondrial fusion protein Mfn2 (Mitofusin 2). Downregulation of Drp1 limits mitochondrial fragmentation, cytochrome c release, caspase activation and apoptosis [101, 107, 109–111]. Adenocarcinomic alveolar epithelial cells, a model of NSCLC, have displayed decreased Drp1 protein expression, which promotes elongation of mitochondrial phenotypes and limits fission while inhibiting the downstream processes of apoptotic activation [112]. Inhibition of fission can also lead to other forms of mitochondrial dysfunction, including loss of mtDNA, increased ROS and ATP depletion [113]. Furthermore, dysfunctional mitochondrial fission in cancer cells directly induces chromosomal instability and centrosome overamplification, which can then initiate the DNA damage response through the modification of the cell cycle to promote survival [114]. These observations suggest the mitochondrial fission machinery could be a putative target to induce apoptosis through overexpression of Drp1 in tumors.

Drp1 function is controlled by post-translational modifications, which have also been shown to influence mitochondrial morphology and subsequent apoptosis [115]. Post-translational modifications of Drp1 influence cell sensitivity to apoptosis by its interaction with Bax/Bak, which is stabilized after mitochondrial fragmentation but prior to cytochrome c release [108]. In other cancer models, ubiquitylation and degradation of Drp1 is important for maintaining mitochondrial biogenesis and metabolic function during interphase of the cell cycle [116]. The implications of Drp1 phosphorylation are perplexing – there seems to be both activating (S585; S616) [117, 118] and inhibiting (S637) [119] Drp1 phosphorylation sites that influence fission. Phosphorylation of Drp1 S585 by cyclin—dependent kinase 1 (Cdk1)/cyclin B during mitosis allows the equal distribution of mitochondria into daughter cells during cytokinesis [118]. Overexpression of cyclin B1, the regulatory subunit for Cdk1, is overexpressed in non-small cell lung cancer and its upregulation is closely associated with poor prognosis [120]. Currently, inhibition of cyclin-dependent kinases, such as Cdk1, is being considered as a therapeutic target to increase the sensitivity of cancer cells to extrinsic cell death [121] and prohibit metastatic processes [122].

If modulation of mitochondrial fission players is a viable therapeutic, then the role of Fis1, MARCH5 and EndoB1 in cancer progression should also be considered. The downregulation of Fis1 inhibits mitochondrial fragmentation and reduces apoptosis [105, 123] while prohibiting Bax translocation and activation [111]. So, Drp1 and Fis1 appear to act at different steps in the mitochondrial fission pathway. Ubiquitin-dependent degradation pathways are connected to cancer promotion because of their integral involvement in protein quality control, signal transduction, and the regulation of immune responses [124], so MARCH5 is another putative target for intervention in cancer. Overexpression of MARCH5 promotes ubiquitination of Drp1 and the formation of elongated, reticular mitochondria in an Mfn2-dependent manner [124]; whereas, mutations in MARCH5 cause fragmentation of the mitochondria [125] which promotes apoptotic initiation. Lastly, Endophilin B1 (EndoB1) [126–128] is a cytosolic protein that transiently interacts with Bax on the mitochondria. EndoB1 binds Bax to promote division of the mitochondrial membrane. EndoB1 knockout mice show increased rate of spontaneous tumor development [129] and downregulation of EndoB1 blocks Bax translocation and cytochrome c release induced by apoptotic stimuli [130]. Manipulation of these fission players greatly impacts the onset of apoptosis.

Mitochondrial fragmentation by the accumulation of fission proteins on the mitochondrial membrane mediates apoptosis. But during Bax foci formation during apoptotic initiation, the compensatory mitochondrial dynamics function, mitochondrial fusion, is blocked [104]. Three large GTPases in the dynamin family mediate mitochondrial fusion: mitofusins 1 and 2 (Mfn1 and Mfn2) and optic atrophy protein 1 (Opa1) [115]. It has been shown that inhibition of mitochondrial fusion promotes apoptosis— (Mfn1/2) silencing promotes mitochondrial fragmentation and increases apoptotic cell death [131]. Evidence has demonstrated that mitofusins are regulated in both post-transcriptional and post-translational fashion to govern their function [132]. A study examining NSCLC patient tissue has identified an alternatively spliced isoform of *Mfn1* that has upregulated expression at the mRNA level [133] that would limit apoptotic action in these cancer cells if protein expression of Mfn1 matched mRNA expression. Mfn2 has also exerted a regulatory role on the cell cycle that impacts cellular proliferation; it was originally named “hyperplasia suppressor gene, HSG” [132]. Mfn2 overexpression limited S and G_2_/M phases while promoting G_0_/G_1_ growth through the binding and sequestration of Ras that ultimately causes cell cycle arrest and inhibits cell proliferation [134].

The anti-proliferative function of mitochondrial fusion proteins is supported by the observation that Opa1 is physically associated with prohibitins (PHB1/2; inner mitochondrial membrane proteins that also regulate the cell cycle and apoptotic processes) to regulate cellular proliferation [135]. However, the loss of Opa1, induces spontaneous apoptosis [136]. It has been shown that Bcl-2 overexpression can prevent cell death caused by Opa1 silencing, which suggests that mitochondrial fusion precedes mitochondrial membrane permeabilization in cell death. Interestingly, silencing Opa1 expression *in vitro* reduced cisplatin resistance, a platinum-containing anti-cancer drug that is given intravenously to treat solid malignancies, and increased the release of cytochrome c from the mitochondria to signal the caspase-dependent apoptotic cascade [137]. Alternatively, overexpression of these mitochondrial fusion proteins elongates the mitochondrial network and inhibits Bax activation, cytochrome c release and apoptosis [138]. Overexpression of Opa1 prevents intrinsic mediated apoptosis, but it does not block apoptosis induced through the extrinsic apoptosis pathway [139]. In lung adenocarcinoma cells, Opa1 is highly expressed and indicates poor prognosis [137].

Pro-apoptotic Bcl-2 family members, Bax and Bak also regulate mitochondrial morphology [96]. In Bax/Bak double knock out cells, mitochondrial fusion is reduced and downregulation of Drp1 in these cells does not promote elongation [96]. This suggests that Bax and Bak activate mitochondrial fusion without disrupting the normal fission process. It is unknown how Bax and Bak promote mitochondrial fusion in healthy cells but remain an integral part of mitochondrial fission. Cancer cells may confer resistance to apoptotic stimuli as a result of a functional loss of proapoptotic proteins and/or increased expression of antiapoptotic proteins.

Some theories suggest that mitochondrial dynamics participate in mitochondrial membrane permeabilization (MMP) - the point of no return during apoptosis [140], during which lipids and components of the fusion/fission machinery act concertedly to remodel mitochondrial membranes [104, 141–143]. Models suggest that upon apoptosis induction EndoB1, Bax, and Drp1 translocate to Mfn2-mitochondrial foci to mediate vesicle scission, removing lipids from the outer mitochondrial membrane. As a result of EndoB1 lipid deformation activity, Bax and Bak induce permeabilization of membranes through pore formation (MOMP; mitochondrial outer membrane permeabilization) which allows for cytochrome c release [142].

Another theory proposes that mitochondrial dynamics proteins regulate cristae remodeling, which favors MMP. Cristae rearrangements are thought to increase apoptotic sensitivity by promoting the release of cytochrome c [144]. Moreover, two pools of cytochrome c have been identified whereby, the major pool is retained in the cristae and binds cardiolipin (an important component of the inner mitochondrial membrane) and the minor, soluble pool of cytochrome c is present in the intermembrane space [144–146]. Release of membrane-bound cytochrome c requires efficient mitochondrial fission [110, 136].

Some controversy still exists regarding the role of mitochondrial dynamics in apoptosis. It has been suggested that various stimuli impact mitochondrial fission and MMP [147], which can profoundly impact the degree of cell death. More studies are required to determine how mitochondrial fragmentation contributes to apoptosis and promotes tumor progression. It is necessary to identify the contributions of mitochondrial dynamics proteins in healthy versus apoptotic cells to clarify the definitive role of mitochondrial dynamics in cancer.

## Conclusions

Mitochondrial function widely impacts dozens of diseases that affect millions of people worldwide. Defects in mitochondrial function are now being acknowledged in the etiology of cancer. Nearly 70 years after the Warburg hypothesis, mitochondrial function is at the crux of cancer biology and therapeutics. Alterations in mitochondrial function have been identified in neoplastic transformation and/or metastasis. A prominent example of mitochondrial dysfunction in cancer is the prominence of mtDNA mutations. As discussed previously, mitochondrial fusion is necessary to maintain the mitochondrial genome. Additionally, since mitochondrial division is also essential for the elimination of damaged mitochondria, any deficits in mitochondrial fission may contribute to the accumulation of dysfunctional mitochondria. It is possible that an imbalance in mitochondrial dynamics could contribute to the loss of mtDNA that is observed in cancer. This information could ultimately be used to develop novel approaches that identify the modification of the activity of affected genes to either prevent or treat cancer. By understanding the alterations in these processes that influence cancer metabolism, more effective treatments can be developed to target specific cancer cells based on their mitochondrial metabolic profile to decrease toxicity or potentially enhance sensitivity of chemotherapeutics.
